# Supplementary material for: Modulation of mTOR signaling as a strategy for the treatment of Pompe disease
Source: EMBO Mol Med. 2017 Jan 27;9(3):353–70. doi: 10.15252/emmm.201606547 (PMC5331267; doi:10.15252/emmm.201606547)
Supplement: Supplementary file 1 — Appendix [file EMMM-9-353-s001.pdf]

Appendix

Modulation of mTOR signaling as a strategy for the treatment of Pompe disease

Jeong-A Lim[1*], Lishu Li[1*], Orian Shirihai[2], Kyle M. Trudeau[2], Rosa Puertollano[3], and Nina Raben[1]

Table of Contents

Reagents……………………………………………………………………………………………………. 2

Appendix Figure S1. Western analysis of KO cell lysates subjected to subcellular fractionation…………… 3

Appendix Figure S2. Immunostaining of WT and KO myotubes with LC3 following starvation in combination with chloroquine …………………………………………………………………………........ 4

Appendix Figure S3. The effect of acNPs on cell viability……………………………………………………… 5

Appendix Figure S4. TSC2 knockdown in KO myotubes……………………………………………………… 6

Appendix Figure S5. Immunostaining of isolated muscle fibers with LAMP1 (red) from AAV1-shRNA-TSC2-infected muscle of a GAA-KO mouse……………………………………………………………….. 7

Reference……………………………………………………………………………………………………... 8

Reagents

The following primary antibodies were used for western blots: anti-4EBP1 (9644), anti-phospho-4E-BP1 (Thr37/46) (9459) and anti-phospho-4E-BP1 (S65) (9451), non-phospho-4EBP1 (Thr46) (4923), eIF4E (2067), eIF4GI (8701), anti-ATF-4 (11815), anti-eIF2α (9722), anti-phospho-eIF2α (9721), anti-phospho-AMPKα-Thr172 (40H9; 2535), anti-AMPKα (D5A2; 2793), anti-LKB1 (D60C5; 3047), anti-AKT (4691), anti-phospho-AKT (Ser473) (4058), anti-PRAS40 (2691), anti-phospho-PRAS40 (Thr246) (2997), anti- S6 Ribosomal Protein (2217), anti-phospho-S6 Ribosomal Protein (Ser235/236) (4858), anti-Tuberin/TSC2 (4308), anti-phospho-Tuberin/TSC2 (Ser1387) (5584), anti- ULK1 (8054) and anti-phospho-ULK1 (Ser757) (6888), anti-Acetyl-CoA Carboxylase (ACC) (3676) and anti-phospho-ACC (Ser79) (11818), anti-mTOR (7C10) (2983) – all from Cell Signaling Technology; rabbit polyclonal anti-eIF4EBP1 (ab2606); anti-PGC1β (ab176328), anti-RHEB (ab25873), anti-COX IV (ab14744), and anti-ATP6V1A, subunit A (V_1) (ab137574) from Abcam; rabbit anti-LC3B (microtubule-associated protein 1 light chain 3) (L7543) and anti-myosin (skeletal slow and fast; M4276 and M8421) from Sigma; rat anti-mouse LAMP-1 (Lysosomal-Associated Membrane Protein 1; 553792) and anti-GFP (8371-2) from BD Pharmingen; anti-Puromycin, clone 12D10 (MABE343) and anti-PGC1α (ST1202) from Millipore; anti-ATP6V0C (Thermo Fisher Scientific; PA5-23972); anti-troponin I-SS (sc-8119) from Santa Cruz Biotechnology. Mouse monoclonal anti-vinculin (Sigma, V 9131), mouse monoclonal anti-GAPDH antibody (Abcam, ab9484) served as loading controls. Alexa Fluor-conjugated secondary anti-mouse (Life Technologies, A-21057) or anti-rabbit (Life Technologies, A-21076) antibodies were used for western blot and immunostaining.

LysoTracker-Red (DND-99; accumulates in endosomes/lysosomes; p*K*a=NA) and LysoSensor Green (DND-189; p*K*a=5.2) [Molecular Probes (L7528 and L7535 respectively)]. Adenovirus expressing either shRNA TSC2 (Ad-GFP-U6-mTSC2; $2.5x10^{10}$PFU/ml) or RHEB (Ad-RFP-mRHEB; $2.8x10^{10}$PFU/ml) was prepared, amplified, and purified by Vector Biolabs (Malvern, PA). rAAV1-shRNA-TSC1/2 was packaged in HEK-293 cells using plasmid pTR-UF11-shRNA-TSC1/2 and helper plasmid pKRAP (titer: $6.00x\ 10^{12}$ vg/ml). AAV vector expressing shRNA-TSC1/2 was prepared and purified at the Powell Gene Therapy Center Vector Core Lab at the University of Florida.

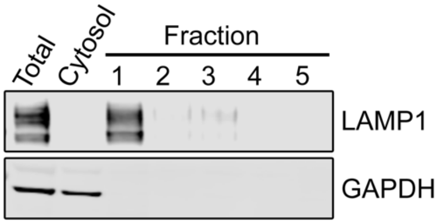

Appendix Figure S1. Western analysis of KO cell lysates subjected to subcellular fractionation. Immunoblotting with cytosolic (GAPDH) and lysosomal (LAMP1) markers was used to confirm an efficient enrichment for lysosomes. KO myotubes (day 9 in differentiation media) were lysed and subjected to fractionation in Percoll gradients as described (Christensen et al, 2003). Briefly, postnuclear supernatants were centrifuged at high speed; high speed supernatants represent "cytosol"; high speed pellets were fractionated in Percoll gradients and five fractions were collected from the bottom of the tube (1.5ml of fraction 1; 1ml of each of the remaining fractions 2-5). Total represents "input". Fraction 1 is enriched in the lysosomes as shown by LAMP1-positive/GAPDH-negative staining. This fraction was used for further analysis. Source data are available online for this figure.

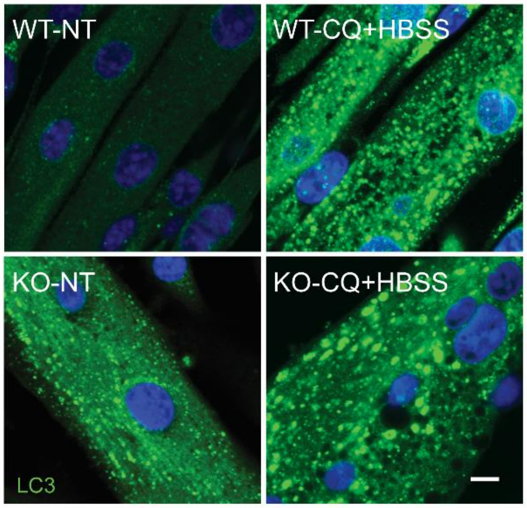

Appendix Figure S2. Immunostaining of WT and KO myotubes with LC3 following a combination of starvation and chloroquine.

WT and KO myotubes were treated with chloroquine (CQ; 6h; 50µM) in combination with starvation (HBSS; 2h); the cells were fixed in acetone/methanol (equal volumes) at -20º C for 15 min, washed in PBS, permeabilized, and immunostained with LC3. As expected, chloroquine (which blocks autophagosomal-lysosomal fusion) in combination with starvation dramatically increased the appearance of LC3-positive structures. The effect is much less obvious in the KO; note that cytosolic staining is prominent in both non-treated and treated KO cells.

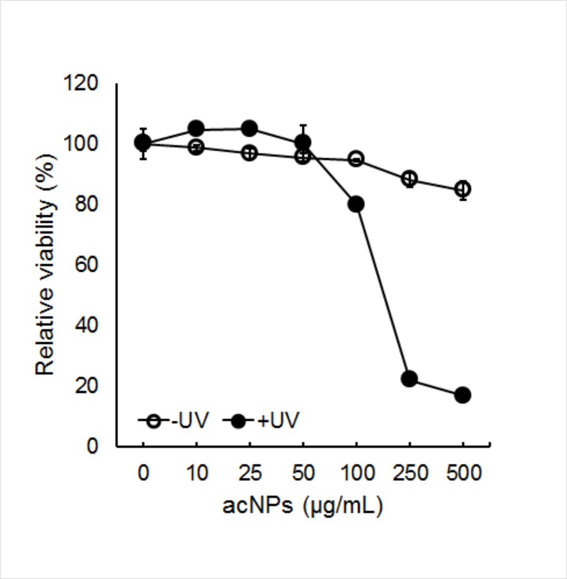

Appendix Figure S3. The effect of acNPs on cell viability.

In a preliminary series of experiments, KO myotubes were exposed to different dosages of acNPs to determine the optimal concentration that doesn't induce cell death. KO myoblasts were incubated with different concentrations of acNPs for 6 hours, followed by exposure to UV-light for 5 min. The cells were then allowed to recover in proliferation medium for 18 hours. Exposure to UV-light for 5 min did not affect cell viability; therefore, cell viability with or without UV-light exposure was taken as 100%. Non-irradiated acNPs-treated cells served as controls. Cell viability was determined using Cell Proliferation Assay Kit (Millipore, 2210). Only minimal cytotoxic effect was observed in cells treated with acNPs alone at concentrations of up to 500 µg/mL. However, a decrease in viability was observed in cells treated with 100 µg/mL of acNPs followed by UV-activation; this cytotoxic effect was much stronger at a concentration of 250 µg/mL. A dose of 50 µg/mL was selected for further studies to attain the highest possible dosage without cytotoxic effect.

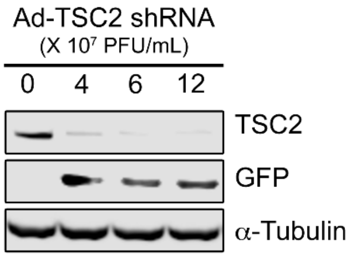

Appendix Figure S4. TSC2 knockdown in KO myotubes.

To determine the optimal concentration of the adenovirus to, KO myotubes were infected with different concentrations of Ad-mTSC2 shRNA for 3 days, and cell lysates were subjected to western analysis with ant-TSC2 and anti-GFP antibodies. Efficient shRNA-mediated knockdown of TSC2 was achieved at 4 x 10^7 PFU/mL. Source data are available online for this figure.

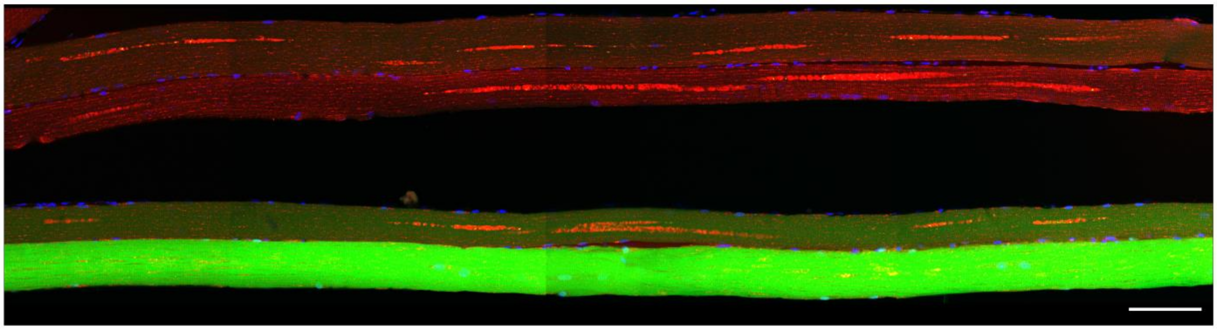

Appendix Figure S5. Montage of confocal fluorescence images of fibers from AAV1-shRNA-TSC2-infected muscle of a GAA-KO mouse. The fibers were stained with LAMP1 (red). Of the four neighboring fibers, only one fiber (bottom) appears efficiently infected (bright green); the infected fiber does not contain clusters of lysosomes which are typically found in the area of autophagic buildup. Bar: 100 μm

Reference

Christensen, E.I., Devuyst, O., Dom, G., Nielsen, R., Van der Smissen, P., Verroust, P., Leruth, M., Guggino, W.B., and Courtoy, P.J. (2003). Loss of chloride channel ClC-5 impairs endocytosis by defective trafficking of megalin and cubilin in kidney proximal tubules. PNAS, 100:8472-7.
