# Supplementary material for: Modulation of mTOR signaling as a strategy for the treatment of Pompe disease
Source: EMBO Mol Med. 2017 Jan 27;9(3):353–70. doi: 10.15252/emmm.201606547 (PMC5331267; doi:10.15252/emmm.201606547)
Supplement: Supplementary file 2 — Expanded View Figures PDF [file EMMM-9-353-s002.pdf]

Expanded View Figures

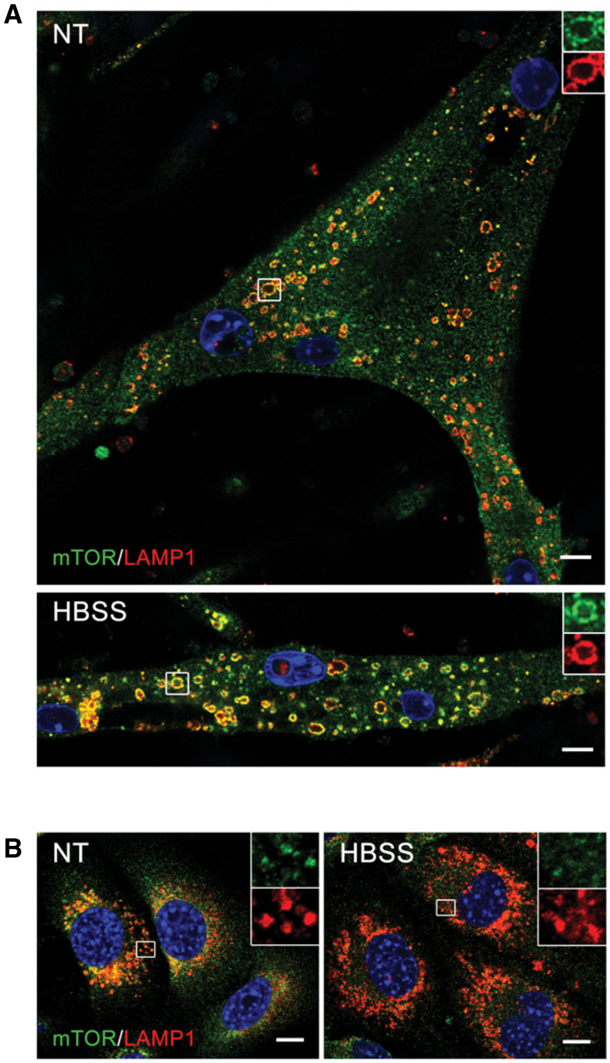

Figure EV1. mTOR remains at the lysosome in starved KO myotubes, but not in myoblasts.

A Lysosomal localization of mTOR after starvation (HBSS) in KO cells was confirmed by using a different immortalized KO cell line (clone RF1). The cells were grown in differentiation medium for 8 days, starved for 2 h, and fixed. The images show immunostaining of fed (NT) and starved (HBSS) KO cells with anti-LAMP1 (red) and anti-mTOR (green) antibodies. Scale bar: 10 μm.

B Immunostaining of KO myoblasts grown in proliferation medium with anti-LAMP1 (red) and anti-mTOR (green) antibodies. Lysosomal localization of mTOR is observed in fully fed KO myoblasts (NT). Almost no co-localization of the two stains is seen in KO myoblasts after 2 h of starvation (HBSS). The images show randomly selected fields of > 100 cells for each condition. Scale bar: 10 μm.

© 2017 The Authors

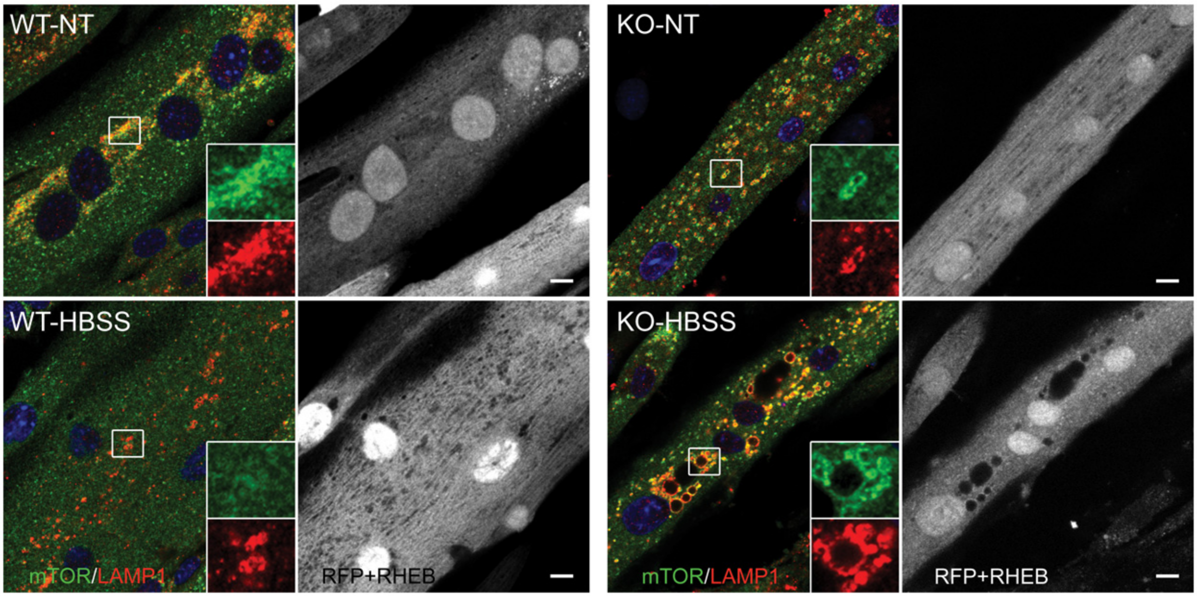

Figure EV2. mTOR remains at the lysosome in fully fed and starved KO cells following RHEB overexpression.
WT and KO myotubes were infected with adenovirus expressing mRHEB (Ad-RFP-mRHEB; RFP is expressed from its own promoter; pseudo white color) for 3 days. The cells were then exposed to starvation (HBSS) for 2 h, fixed, and immunostained with anti-LAMP1 (pseudo red color) and anti-mTOR (green) antibodies. Similar to the data in Fig 6, lysosomal localization of mTOR is observed in both fed and starved KO cells. Scale bar: 10 μm.

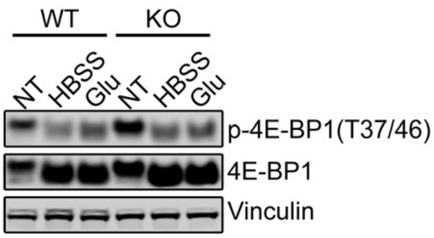

Figure EV3. Effect of glucose starvation on 4E-BP1 phosphorylation in KO cells.
Immunoblot analysis of WT and KO cell lysates after HBSS (2 h) or glucose (Glu) starvation (2 h) with anti-4E-BP1 antibodies. The degree of 4E-BP1 dephosphorylation in WT and KO is different after HBSS treatment, but similar after Glu starvation.

Source data are available online for this figure.

© 2017 The Authors

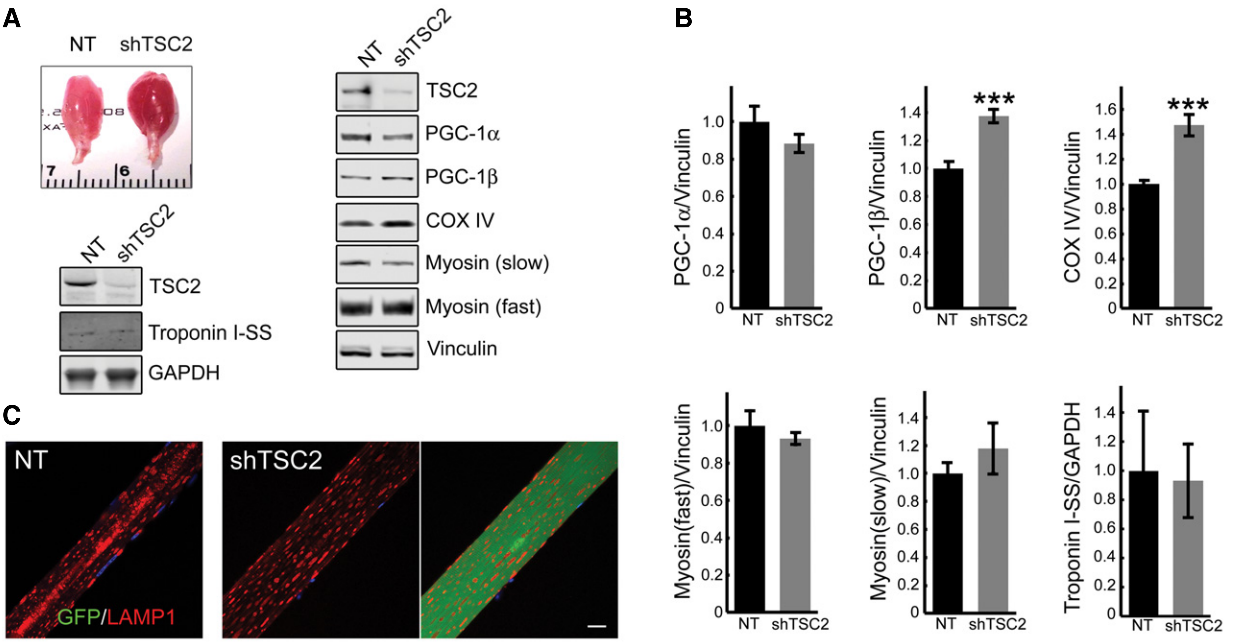

Figure EV4. Characterization of the AAV1-shRNA-TSC2-infected muscle in GAA-KO mice.

A, B Appearance of the infected muscle (reddish color). Immunoblot analysis of sham-infected (NT) and infected (shTSC2) muscle with the indicated antibodies; an increase in the levels of PGC-1β (n = 7) and COX IV (n = 7) is seen in the infected muscle. Graphical representation of the data from Western blots. Data are mean ± SE; Student's t-test; ***$P < 0.001$. The difference in the levels of PGC-1α (n = 7), myosin fast, myosin slow, and troponin (n = 4 each) between non-infected and infected muscle is not significant.

C Immunostaining of sham-infected (left panel) and infected (two right panels) fibers with LAMP1 (red); no major difference in the distribution of lysosomes is observed between the infected and sham-infected fibers; the difference is in the absence of the autophagic area and the size of the infected fiber. Scale bar: 20 μm.

Source data are available online for this figure.

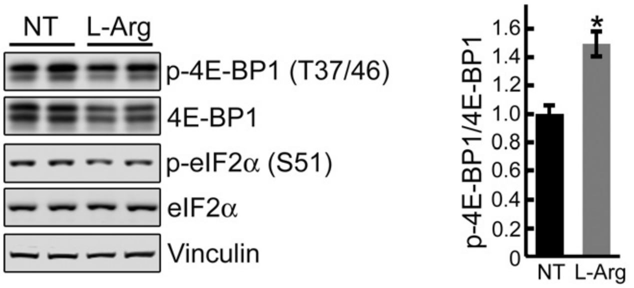

Figure EV5. Effect of L-arginine on 4E-BP1 and eIF2α in KO cells.
Immunoblot analysis showing the effect of arginine treatment (5 mM) in KO myotubes. NT, not treated. Arginine was added to KO cells grown in differentiation medium on days 4, 5, 6, and 7; 15 min after last arginine addition (day 7), cells were lysed and immunoprobed with the indicated antibodies. Vinculin was used as a loading control. Of note, arginine treatment (5 mM) reduced the level of total 4E-BP1 and decreased the levels of p-eIF2α. A decrease in the amount of total 4E-BP1 without much change in the p-4E-BP1 leads to an increase in the p-EBP1$^{T37/46}$/total ratio, indicating mTOR activation. Data are mean ± SE; Student's t-test; *$P < 0.05$; n = 3.

Source data are available online for this figure.

© 2017 The Authors
