# Supplementary material for: Modulation of mTOR signaling as a strategy for the treatment of Pompe disease
Source: EMBO Mol Med. 2017 Jan 27;9(3):353–70. doi: 10.15252/emmm.201606547 (PMC5331267; doi:10.15252/emmm.201606547)
Supplement: Supplementary file 3 — Table EV1 [file EMMM-9-353-s003.docx]

**Table EV1. Actual *P*-values indicated as “significant” in the figures.**

| **Figure** | **Group A** | **Group B** | **p-value** | **Indication** |
| --- | --- | --- | --- | --- |
| **1B** | **WT** | **KO** | **0.00091512** | ******* |
| **2C** | **WT, p-eIF2α/eIF2α** | **KO, p-eIF2α/eIF2α** | **0.02060173** | ***** |
| **2C** | **WT, ATF4** | **KO, ATF4** | **0.02396369** | ***** |
| **2D** | **WT** | **KO** | **0.00157028** | ****** |
| **2E** | **WT, p-S6K/S6K** | **KO, p-S6K/S6K** | **0.03404784** | ***** |
| **2E** | **WT, p-S6/S6** | **KO, p-S6/S6** | **0.01251652** | ***** |
| **3A** | **WT, p-AMPKα** | **KO, p-AMPKα** | **0.04819262** | ***** |
| **3A** | **WT, TSC2** | **KO, TSC2** | **0.00309715** | ****** |
| **3A** | **WT, p-ACC/ACC** | **KO, p-ACC/ACC** | **0.01339475** | ***** |
| **3B** | **WT, p-AMPKα** | **KO, p-AMPKα** | **0.00248980** | ****** |
| **3B** | **WT, TSC2** | **KO, TSC2** | **0.00713051** | ****** |
| **3B** | **WT, LKB1** | **KO, LKB1** | **0.02200538** | ***** |
| **3B** | **WT, RHEB** | **KO, RHEB** | **0.23769057** | none |
| **4B** | **WT, p-4E-BP1/4E-BP1** | **KO, p-4E-BP1/4E-BP1** | **0.00277922** | ****** |
| **4B** | **WT, p-S6/S6** | **KO, p-S6/S6** | **0.00892759** | ****** |
| **4B** | **WT, p-PRAS40/PRAS40** | **KO, p-PRAS40/PRAS40** | **0.00018278** | ******* |
| **4B** | **WT, p-AMPKα** | **KO, p-AMPKα** | **0.03456123** | ***** |
| **4B** | **WT, LKB1** | **KO, LKB1** | **0.009708** | ****** |
| **4B** | **WT, p-TSC2/TSC2** | **KO, p-TSC2/TSC2** | **0.03234699** | ***** |
| **4C** | **WT** | **KO** | **0.03924913** | ***** |
| **4E** | **WT, mTORC1** | **KO, mTORC1** | **0.00021029** | ******* |
| **4E** | **WT, TSC2** | **KO, TSC2** | **0.00466914** | ****** |
| **4E** | **WT, AMPKα** | **KO, AMPKα** | **0.00467516** | ****** |
| **4E** | **WT, RHEB** | **KO, RHEB** | **0.06296995** | none |
| **5A** | **WT, p-4E-BP1/4E-BP1 HBSS 2h** | **KO, p-4E-BP1/4E-BP1 HBSS 2h** | **0.00266481** | ****** |
| **5A** | **WT, p-S6/S6 HBSS 2h** | **KO, p-S6/S6 HBSS 2h** | **0.01215443** | ***** |
| **5B** | **WT, HBSS 2h** | **WT, HBSS+Serum 2h** | **0.76867934** | n.s. |
| **5B** | **KO, HBSS 2h** | **KO, HBSS+Serum 2h** | **0.02979248** | ***** |
| **5D** | **WT, p-ULK1/vinculin** | **KO, p-ULK1/vinculin** | **0.00026526** | ******* |
| **5D** | **WT,** **p-ULK1/vinculin HBSS 2h** | **KO, p-ULK1/vinculin HBSS 2h** | **0.01457861** | ***** |
| **5D** | **WT,** **p-ULK1/vinculin** | **WT,** **p-ULK1/vinculin HBSS 2h** | **0.00259294** | none |
| **5D** | **KO,** **p-ULK1/vinculin** | **KO, p-ULK1/vinculin HBSS 2h** | **0.00451431** | none |
| **5D** | **WT, LC3-II/total** | **WT, LC3-II/total HBSS 2h** | **0.00088448** | ******* |
| **5D** | **KO, LC3-II/total** | **KO, LC3-II/total HBSS 2h** | **0.04899741** | ***** |
| **5D** | **WT, LC3-II/total** | **KO, LC3-II/total** | **0.04316132** | none |
| **5D** | **WT, LC3-II/total HBSS 2h** | **WT, LC3-II/total HBSS 2h** | **0.07344737** | none |
| **6B** | **WT, mTOR** | **WT, mTOR HBSS** | **0.00216136** | ****** |
| **6B** | **WT, mTOR** | **KO, mTOR** | **0.02492888** | ***** |
| **6C** | **WT, TSC2** | **WT, TSC2 HBSS 2h** | **0.01338156** | ***** |
| **6C** | **WT, TSC2** | **KO, TSC2** | **0.01308573** | ***** |
| **6C** | **KO, TSC2** | **KO, TSC2 HBSS 2h** | **0.04689187** | ***** |
| **6C** | **WT, AMPKα** | **WT, AMPKα HBSS 2h** | **0.02463852** | ***** |
| **6C** | **WT, AMPKα** | **KO, AMPKα** | **0.00339237** | ****** |
| **6C** | **KO, AMPKα** | **KO, AMPKα HBSS 2h** | **0.00377952** | ****** |
| **7B** | **KO, V0/V1** | **KO, V0/V1 Glu** | **0.00417900** | ****** |
| **9B** | **KO** | **KO, acNPs** | **0.00000032** | ******* |
| **9D** | **KO** | **KO, acNPs+HBSS** | **0.03349059** | ***** |
| **9D** | **KO, HBSS** | **KO, acNPs+HBSS** | **0.00313126** | ****** |
| **9D** | **KO, acNPs** | **KO, acNPs+HBSS** | **0.02366343** | ***** |
| **10B** | **KO, TSC2/vinculin** | **KO, TSC2/vinculin, shTSC2** | **1.60972E-07** | ******* |
| **10B** | **KO, p-4E-BP1^S65^/4E-BP1** | **KO, p-4E-BP1^S65^/4E-BP1, shTSC2** | **0.00021337** | ******* |
| **10B** | **KO, p-4E-BP1^T37/46^/4E-BP1** | **KO, p-4E-BP1^T37/46^/4E-BP1, shTSC2** | **0.04609856** | ***** |
| **10B** | **KO, p-S6/S6** | **KO, p-S6/S6, shTSC2** | **0.000986004** | ******* |
| **10B** | **KO, p-AKT/AKT** | **KO, p-AKT/AKT, TSC2** | **9.68101E-06** | ******* |
| **10B** | **KO, p-PRAS40/PRAS40** | **KO, p-PRAS40/PRAS40, TSC2** | **0.000922953** | ******* |
| **10C** | **KO** | **KO, shTSC2** | **7.47548E-07** | ******* |
| **10D** | **WT** | **KO** | **2.93E-51** | ******* |
| **10D** | **KO** | **KO. shTSC2** | **1.47E-47** | ******* |
| **10E** | **KO** | **KO, shTSC2** | **2.59777E-09** | ******* |
| **10F** | **KO, p-ULK1^S757^/ULK1** | **KO, p-ULK1^S757^/ULK1, shTSC2** | **0.00016977** | ******* |
| **10F** | **KO, LC3-II/vinculin** | **KO, LC3-II/vinculin, shTSC2** | **0.003280065** | ****** |
| **11B** | **KO** | **KO, L-Arg** | **0.018724637** | ***** |
| **11C** | **KO, p-4E-BP1^S65^/4E-BP1** | **KO, p-4E-BP1^S65^/4E-BP1, L-Arg** | **0.002296475** | ****** |
| **11C** | **KO, p-4E-BP1^T37/46^/4E-BP1** | **KO, p-4E-BP1^T37/46^/4E-BP1, L-Arg** | **0.027307107** | ***** |
| **EV4 B** | **KO, PGC-1α/vinculin** | **KO, PGC-1α/vinculin** | **0.280522693** | none |
| **EV4 B** | **KO, PGC-1β/vinculin** | **KO, PGC-1β/vinculin, shTSC2** | **0.000226695** | ******* |
| **EV4 B** | **KO, COXIV/vinculin** | **KO, COXIV/vinculin, shTSC2** | **0.000304923** | ******* |
| **EV4 B** | **KO myosin fast/vinculin** | **KO myosin fast/vinculin, shTSC2** | **0.603313803** | none |
| **EV4 B** | **KO myosin slow/vinculin** | **KO myosin slow/vinculin, shTSC2** | **0.56195972** | none |
| **EV4 B** | **KO troponin/GAPDH** | **KO troponin/GAPDH, shTSC2** | **0.878138463** | none |
| **EV5** | **KO** | **KO, L-Arg** | **0.018617133** | ***** |
