# Supplementary figures and images for: Modulation of mTOR signaling as a strategy for the treatment of Pompe disease
Source: EMBO Mol Med. 2017 Jan 27;9(3):353–70. doi: 10.15252/emmm.201606547 (PMC5331267; doi:10.15252/emmm.201606547)

Source data for Appendix Fig S1

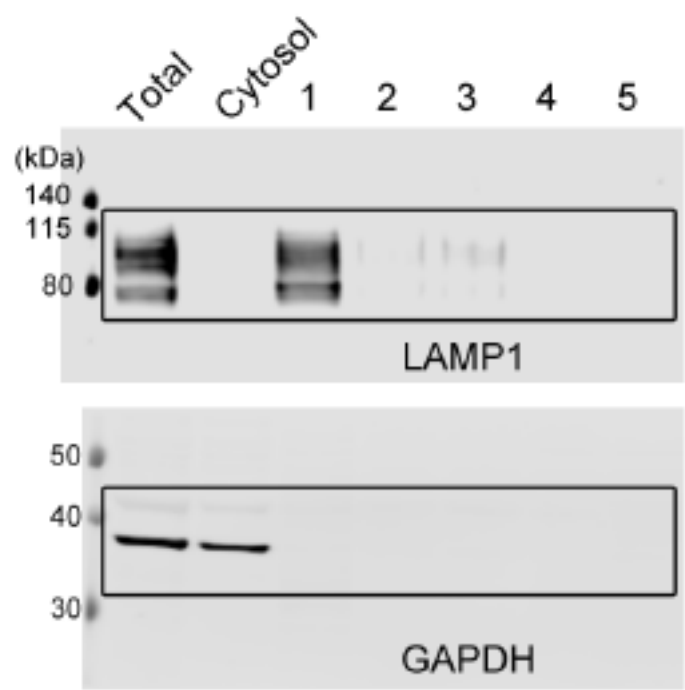

Supplement: Supplementary file 4 — Source Data for Expanded View and Appendix [file EMMM-9-353-s004.zip › Source_Data_For_Appendix+EV_figures/Source_Data_For_AppendixFigureS1.pdf]

Source data for Appendix Fig S4

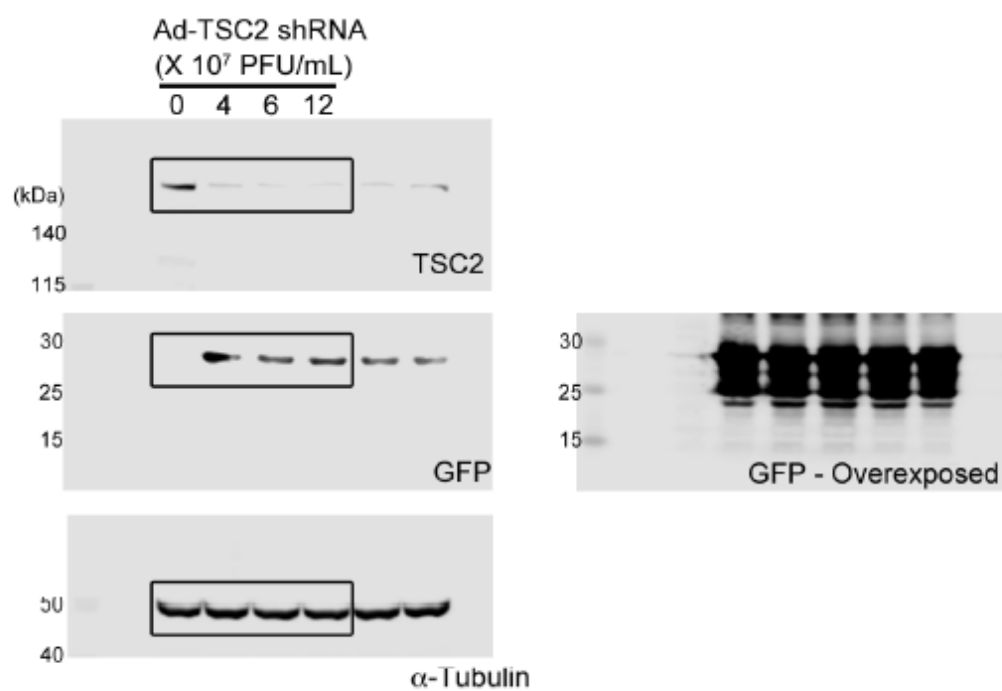

Supplement: Supplementary file 4 — Source Data for Expanded View and Appendix [file EMMM-9-353-s004.zip › Source_Data_For_Appendix+EV_figures/Source_Data_For_AppendixFigureS4.pdf]

Source data for Figure EV3

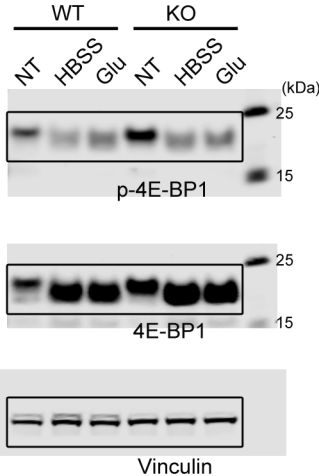

Supplement: Supplementary file 4 — Source Data for Expanded View and Appendix [file EMMM-9-353-s004.zip › Source_Data_For_Appendix+EV_figures/Source_Data_For_FigureEV3.pdf]

Source data for Figure EV4

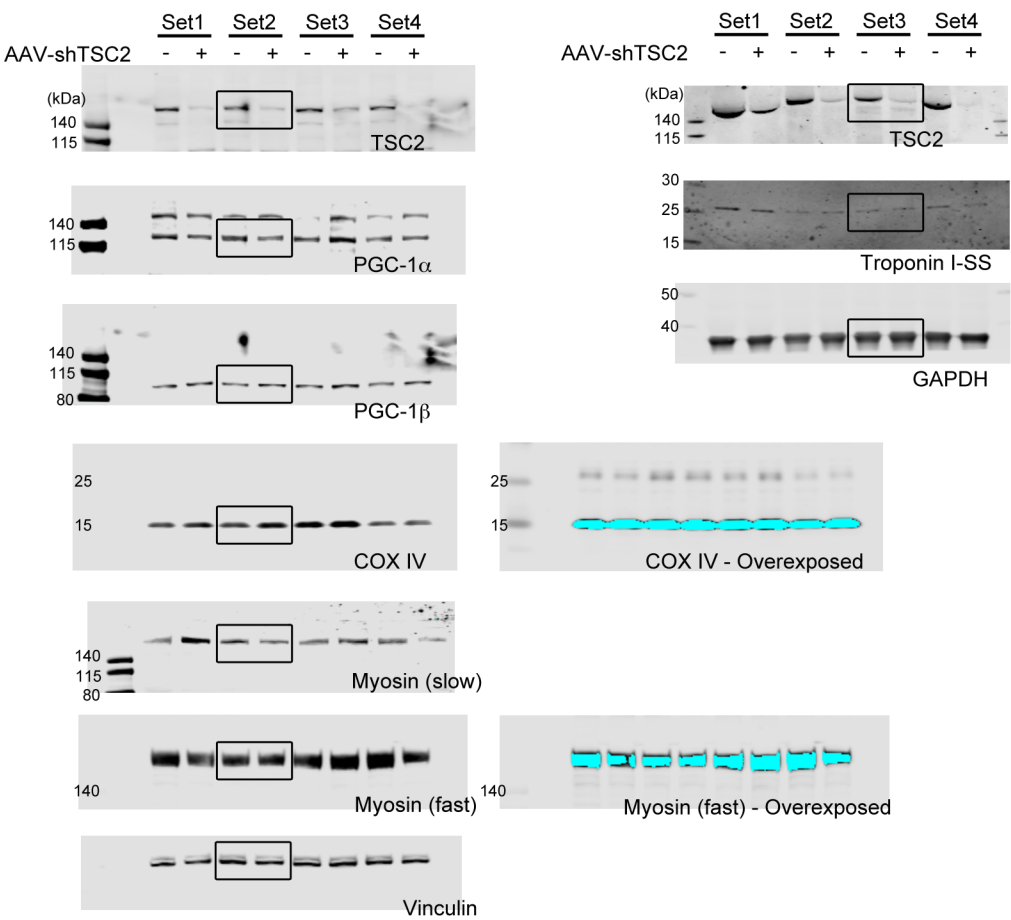

These 4 sets are the same as those shown in source data for Figure 10A.

Supplement: Supplementary file 4 — Source Data for Expanded View and Appendix [file EMMM-9-353-s004.zip › Source_Data_For_Appendix+EV_figures/Source_Data_For_FigureEV4.pdf]

Source data for Figure EV5

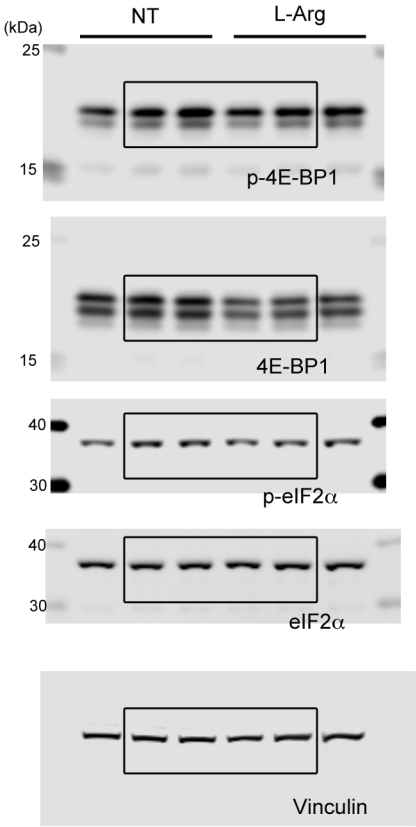

Supplement: Supplementary file 4 — Source Data for Expanded View and Appendix [file EMMM-9-353-s004.zip › Source_Data_For_Appendix+EV_figures/Source_Data_For_FigureEV5.pdf]

Source data for Figure 1

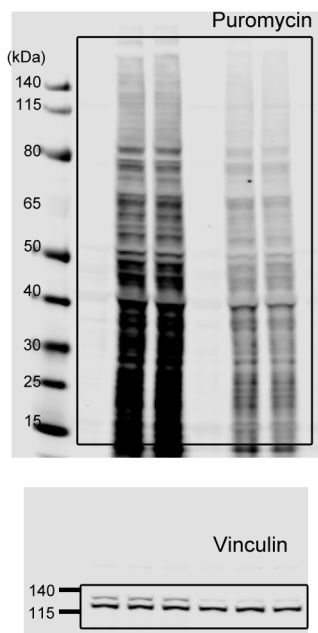

Supplement: Supplementary file 6 — Source Data for Figure 1 [file EMMM-9-353-s005.pdf]

Source data for Figure 2

2A

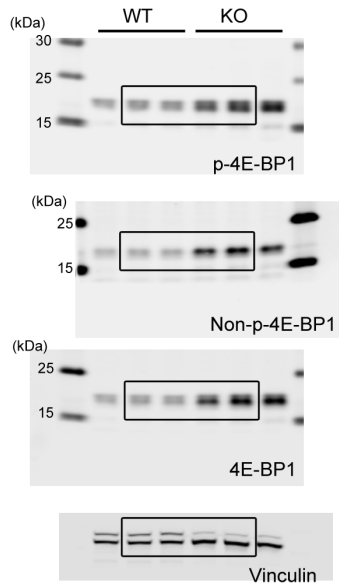

2B

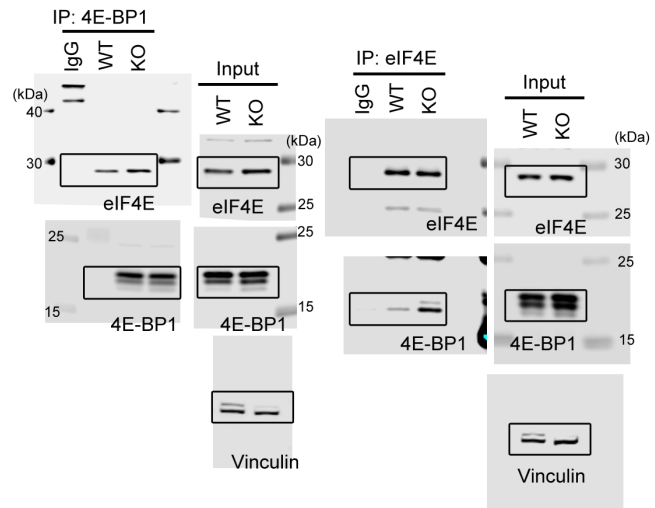

2C

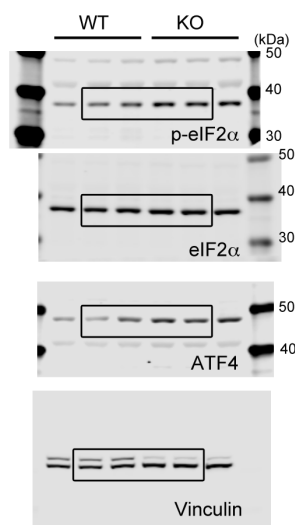

2D

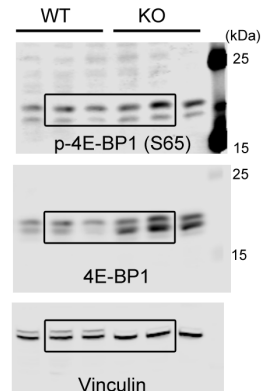

2E

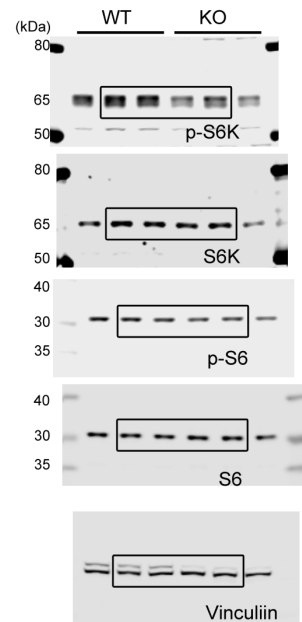

Supplement: Supplementary file 7 — Source Data for Figure 2 [file EMMM-9-353-s006.pdf]

Source data for Figure 3

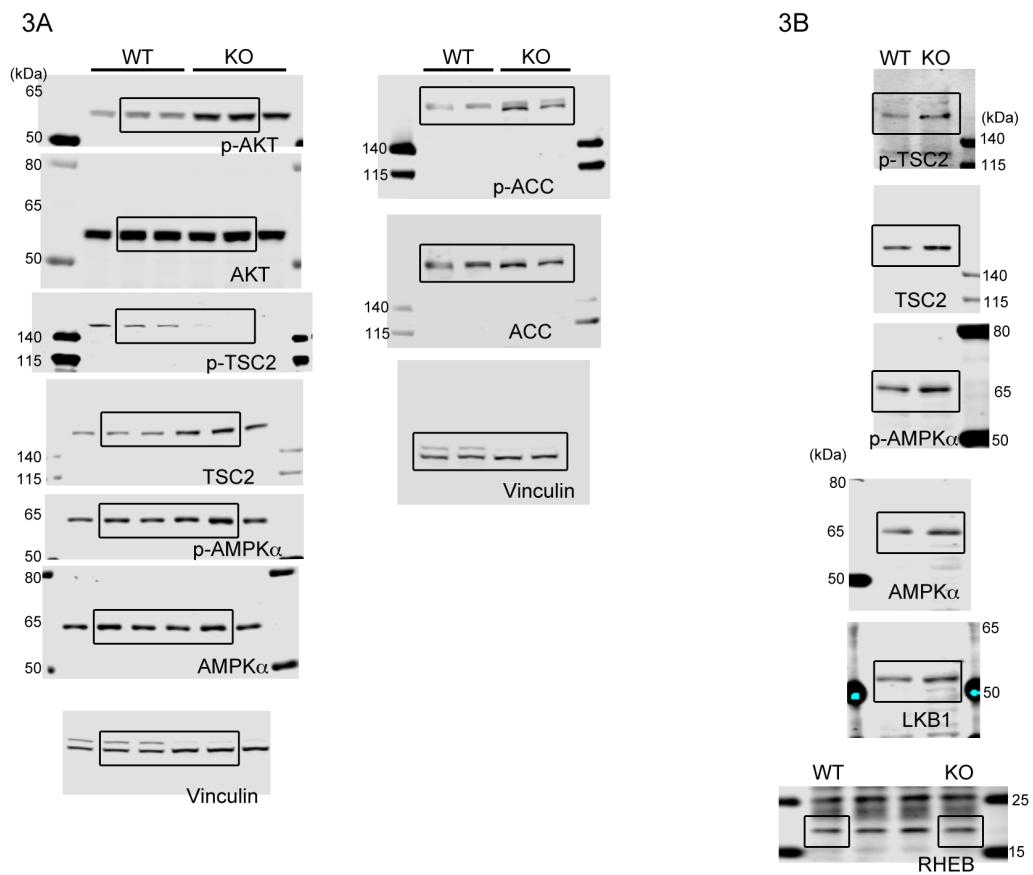

Supplement: Supplementary file 8 — Source Data for Figure 3 [file EMMM-9-353-s007.pdf]

Source data for Figure 4

4A

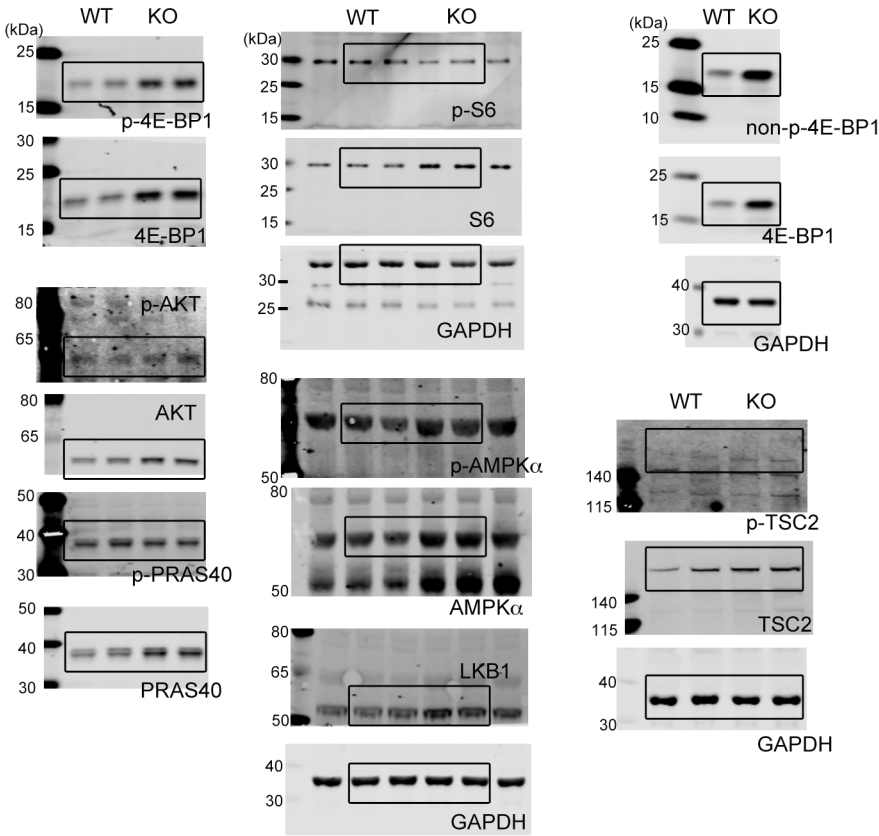

4D

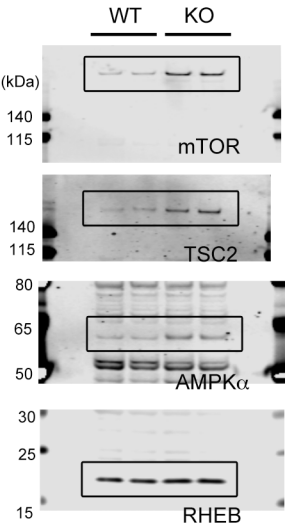

Supplement: Supplementary file 9 — Source Data for Figure 4 [file EMMM-9-353-s008.pdf]

Source data for Figure 5

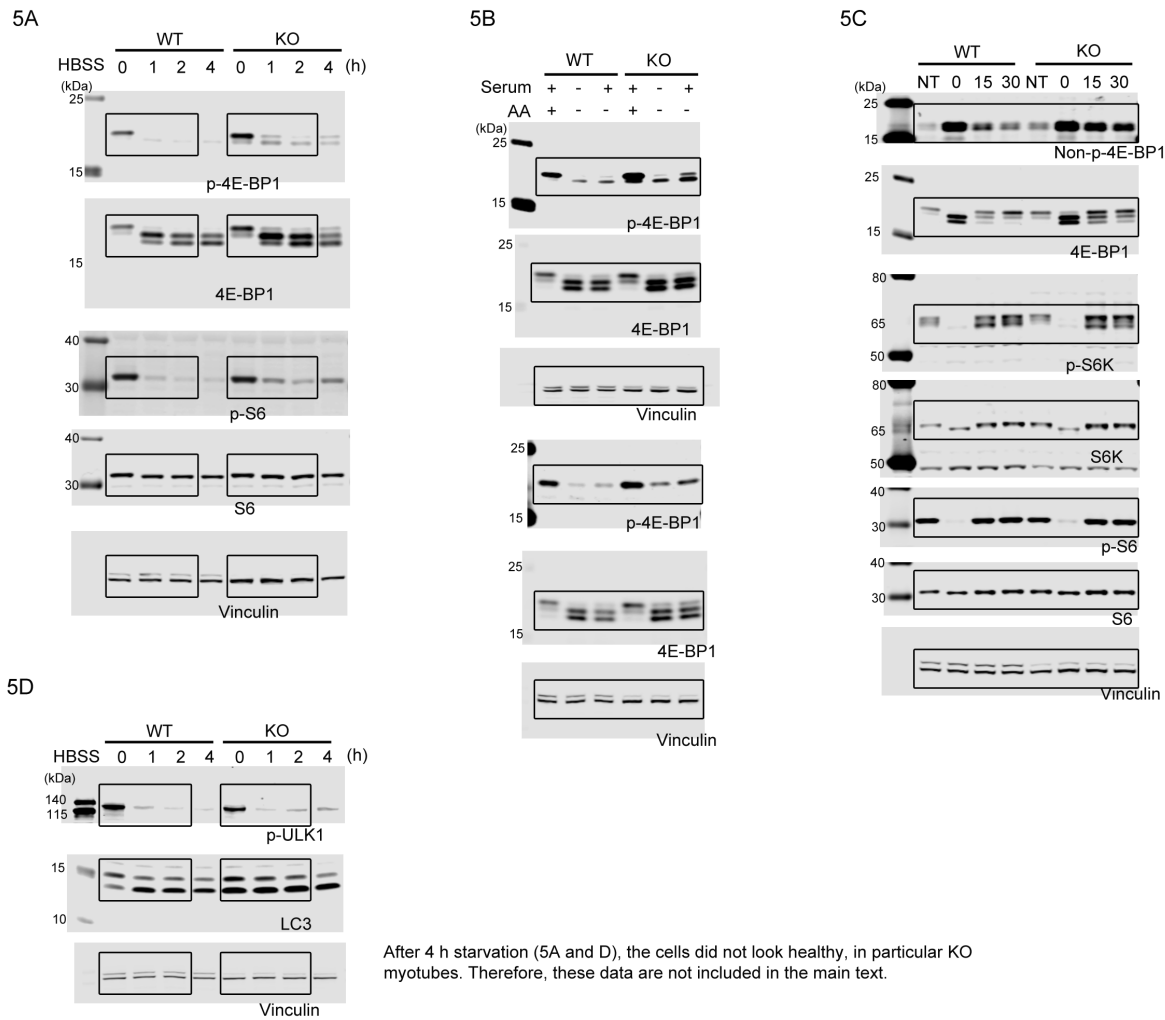

Supplement: Supplementary file 10 — Source Data for Figure 5 [file EMMM-9-353-s009.pdf]

# Source data for Figure 6B

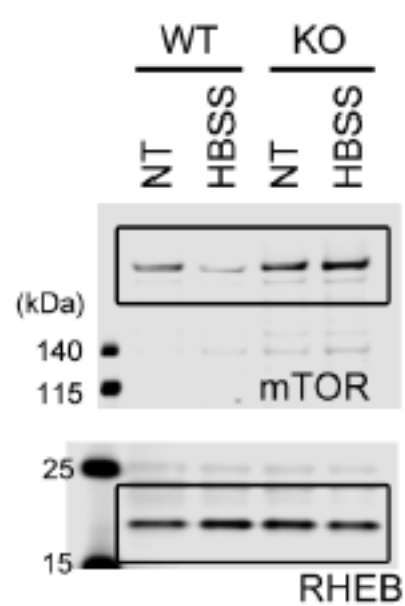

Supplement: Supplementary file 11 — Source Data for Figure 6 [file EMMM-9-353-s010.pdf]

Source data for Figure 7B

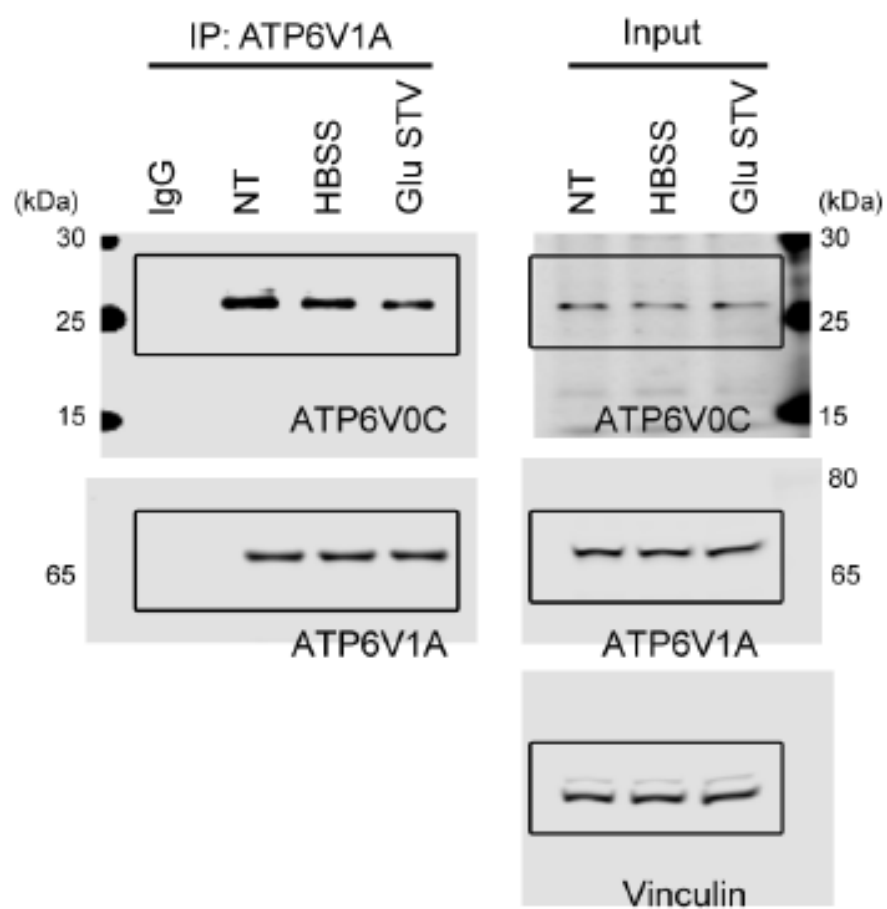

Supplement: Supplementary file 12 — Source Data for Figure 7 [file EMMM-9-353-s011.pdf]

Source data for Figure 10

10A

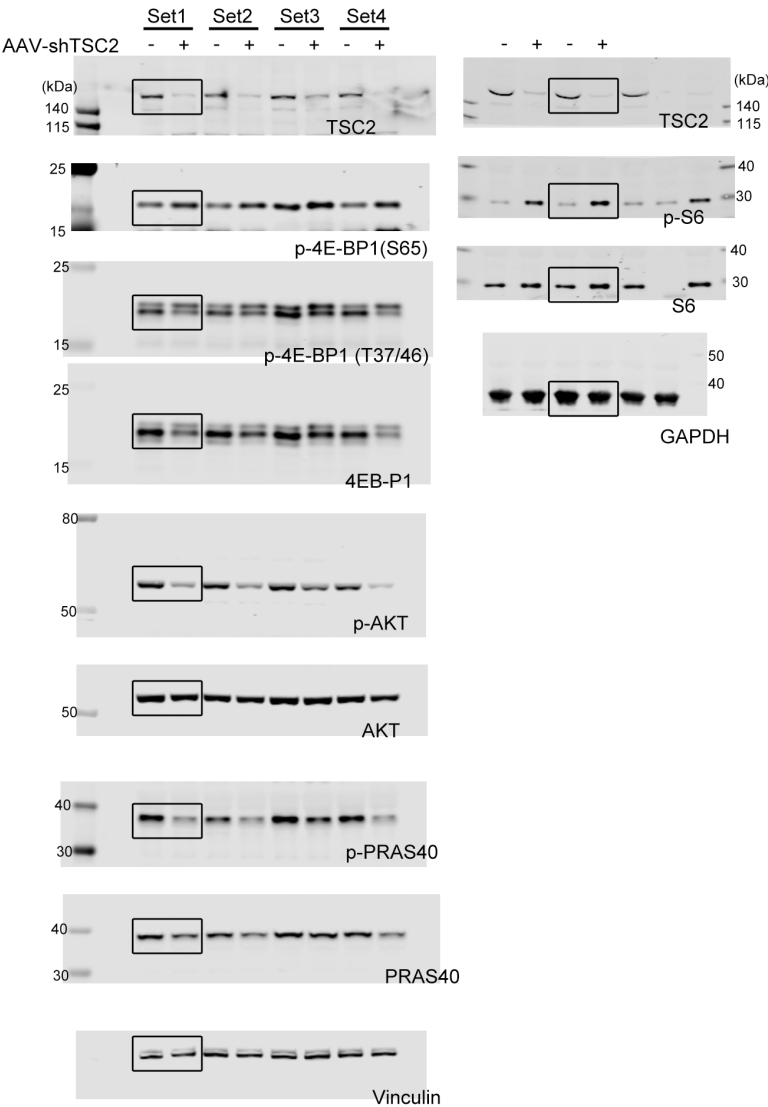

10F

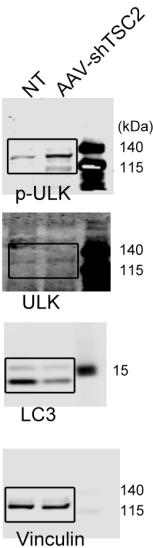

Supplement: Supplementary file 14 — Source Data for Figure 10 [file EMMM-9-353-s013.pdf]

Source data for Figure 11

11A

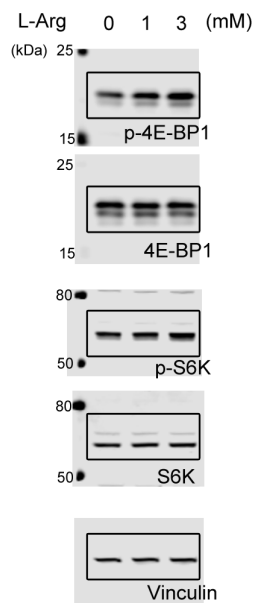

11B

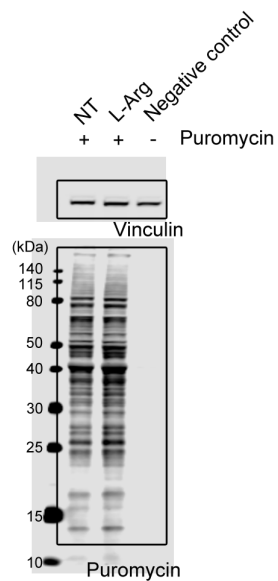

11C

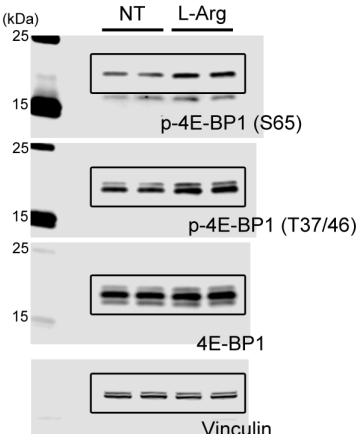

Supplement: Supplementary file 15 — Source Data for Figure 11 [file EMMM-9-353-s014.pdf]
