# Supplementary material for: Modulation of mTOR signaling as a strategy for the treatment of Pompe disease
Source: EMBO Mol Med. 2017 Jan 27;9(3):353–70. doi: 10.15252/emmm.201606547 (PMC5331267; doi:10.15252/emmm.201606547)

Source data for Figure 9

9E

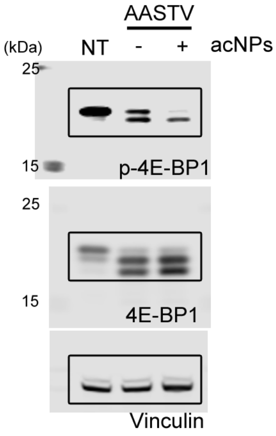

9F

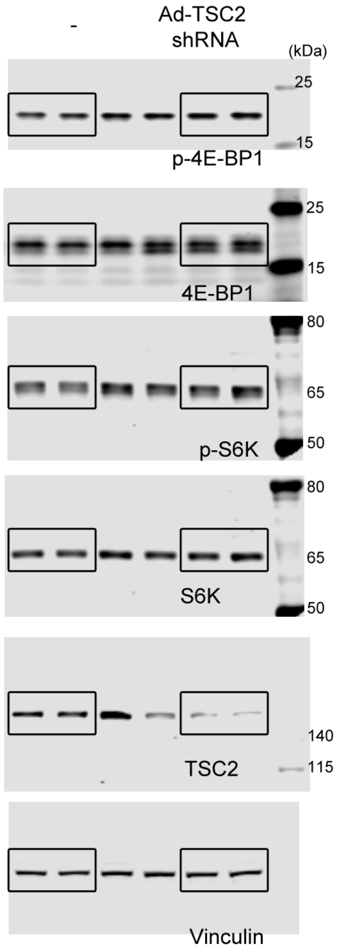

Lanes 1 and 2 were selected in 9F because they show consistent levels of TSC2. Lanes 5 and 6 were selected because they exhibit efficient TSC2 knockdown.

9G

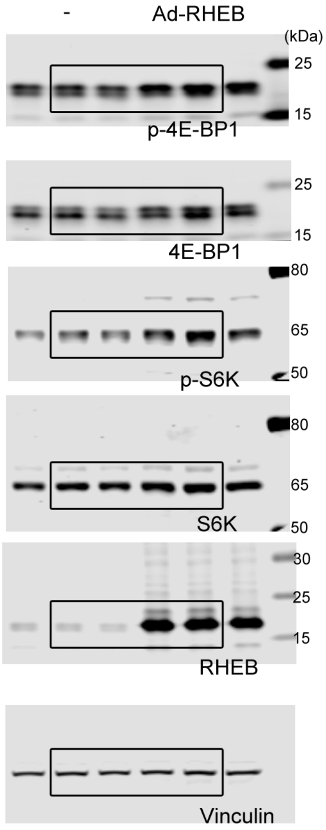

Supplement: Supplementary file 13 — Source Data for Figure 9 [file EMMM-9-353-s012.pdf]
